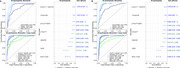# Combining Lumipulse pTau217 and Aβ42 as confirmatory tests for Aβ pathology prior to DMT

**DOI:** 10.1002/alz70856_104702

**Published:** 2026-01-07

**Authors:** James D. Doecke, Ahmed Chenna, Mintzu Lo, Youssouf Badal, Brandon Yee, Robert L. Martone, Christos Petropoulos, Christopher J Fowler, Simon M. Laws, Stephanie R Rainey‐Smith, Ralph N Martins, Christopher C. Rowe, Colin L Masters, John W Winslow

**Affiliations:** ^1^ Collaborative Genomics and Translation Group, School of Medical and Health Sciences, Edith Cowan University, Joondalup, Western Australia, Australia; ^2^ The Australian e‐Health Research Centre, CSIRO, Brisbane, QLD, Australia; ^3^ Monogram Biosciences/Labcorp, South San Francisco, CA, USA; ^4^ Labcorp‐Monogram Biosciences, South San Francisco, CA, USA; ^5^ Labcorp Drug Development, Indianapolis, IN, USA; ^6^ The Florey Institute of Neuroscience and Mental Health, The University of Melbourne, Parkville, Melbourne, VIC, Australia; ^7^ Collaborative Genomics and Translation Group, Edith Cowan University, Joondalup, Western Australia, Australia; ^8^ Centre for Precision Health, Edith Cowan University, Joondalup, Western Australia, Australia; ^9^ Australian Alzheimer's Research Foundation, Perth, Western Australia, Australia; ^10^ Centre for Healthy Ageing, Murdoch University, Murdoch, Western Australia, Australia; ^11^ School of Psychological Science, University of Western Australia, Crawley, Western Australia, Australia; ^12^ School of Medical and Health Sciences, Edith Cowan University, Joondalup, Western Australia, Australia; ^13^ School of Medical and Health Sciences, Edith Cowan University, Perth, Western Australia, Australia; ^14^ Department of Biomedical Sciences, Macquarie University, Macquarie Park, NSW, Australia; ^15^ Molecular Research and Therapy, Austin Health and University of Melbourne, Heidelberg, VIC, Australia; ^16^ Florey Department of Neuroscience and Mental Health, University of Melbourne, Parkville, VIC, Australia

## Abstract

**Background:**

With the increasing number of countries approving disease‐modifying therapies (DMTs) for patients with either Mild Cognitive Impairment (MCI) or mild Alzheimer's disease (AD), it is vitally important to streamline treatment assessment processes. Blood‐based biomarkers (BBMs) have been suggested as rivals to cerebrospinal fluid (CSF) biomarkers in their accuracy to detect neocortical Amyloid‐Beta (Aβ). However, there is little consensus on potential thresholds and resulting confirmatory test performance for international use in target populations.

**Method:**

Two separate sub‐cohorts—the AD continuum cohort (ADCC) [cognitively impaired + unimpaired; *N* = 197] and the intention to treat cohort (ITTC) [cognitively impaired; *N* = 200]—from the Australian Imaging Biomarkers and Lifestyle (AIBL) study of aging, were designed to test the accuracy and potential cut‐offs of leading BBM Lumipulse assays from Fujirebio (pTau217 and Aβ42/40) to detect PET‐Aβ (centiloid ≥25; amyloid prevalence ∼63%).

**Result:**

Using the pTau217/Aβ42 ratio significantly improved the area under the curve (AUC) over pTau217 alone to detect PET‐Aβ positivity in both the ADCC and ITTC (Figure 1A, ADCC *p* = 0.01; Figure 1B: ITTC *p* = 0.009). The Youden's Index cut‐off for pTau217 was higher in the ITTC compared to the ADCC (0.25 pg/mL vs. 0.179 pg/mL). The highest accuracy observed for either single BBMs, the ratio of BBMs, or the linear combination of BBMs that included age, gender, and *APOE* ε4 was 93‐95% in the ADCC (linear combination of pTau217, Aβ42/40, age, gender, and *APOE* ε4; pTau217/Aβ42 ratio) and 95‐97% in the ITTC ( linear combination; pTau217/Aβ42 ratio). The lowest number of participants in the intermediate zone using dual cut‐offs at 95% sensitivity and specificity was 9% and 14% for the pTau217/Aβ42 ratio in the ADCC and ITTC (92‐93% accuracy), and 0% for the linear combination (pTau217, Aβ42/40, age, gender, and *APOE* ε4) in the ITTC (95% accuracy).

**Conclusion:**

The general performance of the Lumipulse assays was similar across both the ADCC and ITTC, indicating strong repeatability independent of clinical stage. Focusing on only participants eligible for DMTs increased sensitivity and improved accuracy for the Aβ‐containing pTau217/Aβ42 ratio and linear combination of markers, and resulted in small numbers of unclassified participants by the test.